# ZFHX3 Knockdown Enhances Metabolic Distress in Atrial Myocytes Through Mitochondrial and Calcium Dysregulation: Mitigation by Trimetazidine

**DOI:** 10.3390/ijms26178576

**Published:** 2025-09-03

**Authors:** Baigalmaa Lkhagva, Shuen-Hsin Liu, Satoshi Higa, Yu-Hsun Kao, Yi-Jen Chen

**Affiliations:** 1 Graduate Institute of Clinical Medicine, College of Medicine, Taipei Medical University, Taipei 11031, Taiwan; bglmaalkh@gmail.com (B.L.); 08787@s.tmu.edu.tw (S.-H.L.); yjchen@tmu.edu.tw (Y.-J.C.); 2 Division of Cardiology, Department of Internal Medicine, Shuang-Ho Hospital, Taipei Medical University, New Taipei City 235, Taiwan; 3 Cardiac Electrophysiology and Pacing Laboratory, Division of Cardiovascular Medicine, Makiminato Central Hospital, 1199 Makiminato, Urasoe 901-2131, Japan; higa@haku-ai.or.jp; 4 Department of Medical Education and Research, Wan Fang Hospital, Taipei Medical University, Taipei 11696, Taiwan; 5 Division of Cardiology, Department of Internal Medicine, Wan-Fang Hospital, Taipei Medical University, Taipei 11696, Taiwan; 6 Cardiovascular Research Center, Wan-Fang Hospital, Taipei Medical University, Taipei 11696, Taiwan

**Keywords:** atrial fibrillation, lactate, mitochondrial Ca^2+^, mitochondrial energy metabolism, trimetazidine, zinc finger homeobox 3 (ZFHX3)

## Abstract

Metabolic dysregulation in the heart plays a critical role in the pathogenesis of atrial fibrillation (AF), yet the underlying molecular mechanisms remain unclear. Loss-of-function variants in the zinc finger homeobox 3 gene (*ZFHX3*) increase AF risk by promoting structural and electrical remodeling. However, the role of *ZFHX3* knockdown (KD) in cardiac metabolism has not been fully elucidated. This study investigated the impact of *ZFHX3* KD on energy metabolism in atrial myocytes and assessed the therapeutic potential of trimetazidine (TMZ). Seahorse XFe24 extracellular flux analysis, bioluminescent assays, microplate enzyme activity assays, and Western blotting were used to study energy substrate (glucose and fatty acid) oxidation stress, intracellular lactate content, glucose uptake, pyruvate dehydrogenase (PDH) activity, and regulatory protein expression in control and *ZFHX3* KD HL-1 cells with or without TMZ (10 μM) treatment. *ZFHX3* KD cells exhibited a higher acute response in oxygen consumption after Etomoxir injection, upregulated CD36 and phosphorylated ACC expression, increased glucose uptake and lactate production, reduced PDH activity, and higher levels of PDK4 and LDHA. Furthermore, *ZFHX3* KD cells showed mitochondrial Ca^2+^ overload and increased phosphorylated PDH and oxidized CaMKII proteins, all of which were significantly attenuated by TMZ. Additionally, TMZ improved mitochondrial dysfunction in *ZFHX3* KD cells by decreasing basal and maximal respiration, spare capacity, and proton leak. These findings suggest that *ZFHX3* downregulation shifts substrate preference toward fatty acid utilization at the expense of glucose oxidation, contributing to metabolic and mitochondrial calcium dysregulation. TMZ mitigates these effects, highlighting its therapeutic potential in AF associated with ZFHX3 deficiency.

## 1. Introduction

Atrial fibrillation (AF) is the most common sustained tachyarrhythmia encountered in clinical practice and a major contributor to cardiovascular morbidity and mortality [1]. Increasing evidence indicates that AF should be recognized not only as an electrical disorder but also as a metabolic cardiac disease [2]. The heart has a high energy demand, and disturbances in substrate flexibility, trafficking, storage, and utilization often precede electrophysiological, contractile, and structural remodeling [3]. Recent proteomic analyses of left atrial appendage tissues from AF patients have revealed alterations in mitochondrial function, oxidative phosphorylation (OXPHOS), sirtuin signaling, the tricarboxylic acid (TCA) cycle, and other key enzymes critical to energy metabolism [4,5]. Metabolomic profiling of blood plasma has also revealed elevated levels of long-chain fatty acids (FAs) and dysregulation of lipid transport proteins [6]. Patients with persistent AF exhibit increased levels of enzymes and metabolites related to ketone body metabolism, along with elevated ketogenic amino acids and glycine [7]. Together, these findings highlight the central role of altered cardiac metabolism in AF pathogenesis and underscore the need to elucidate the molecular mechanisms of atrial metabolic remodeling.

More than a decade ago, multi-ethnic genome-wide association studies identified variants in the zinc finger homeobox 3 (*ZFHX3*) gene on chromosome 16q22 as being strongly associated with AF [8,9,10,11]. Subsequent studies showed that *ZFHX3* plays a significant role in AF pathophysiology [12,13,14]. Cardiac-specific loss of *ZFHX3* in mice increases AF susceptibility, accompanied by slowed conduction velocity, dysregulated calcium handling, atrial enlargement, thrombus formation, and dilated cardiomyopathy [15]. Transcriptomic analyses have revealed that oxidative phosphorylation, mitochondrial organization, and FAs metabolism pathways are downregulated in *ZFHX3*-deficient atria, while inflammatory and injury-related genes are upregulated [15]. These findings suggest that *ZFHX3* variants predominantly exert loss-of-function effects, disrupting both atrial electrophysiology and metabolism. Taken together, these findings indicate that *ZFHX3* plays a critical role in atrial metabolism and calcium homeostasis. However, the role of *ZFHX3* in cardiac energy substrate regulation remains poorly defined, and therapeutic interventions targeting metabolic remodeling that could mitigate these effects have not been established. In this study, we investigated the impact of *ZFHX3* knockdown (KD) on mitochondrial energy metabolism and calcium homeostasis in atrial myocytes and evaluated the protective effects of the metabolic modular trimetazidine (TMZ). Our findings provide mechanistic insight into how ZFHX3 deficiency contributes to metabolic stress in AF and suggest potential therapeutic avenues.

## 2. Results

### 2.1. Effects of ZFHX3 Gene on Mitochondrial Energy Substrate Oxidation

**Figure 1:**
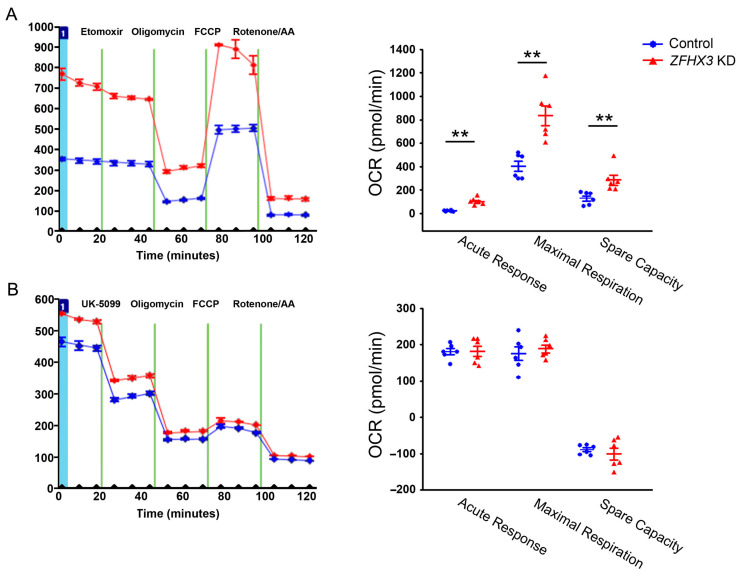
Effects of *ZFHX3* knockdown on fatty acids (FAs) and glucose substrate utilization. Representative traces and quantified data of oxygen consumption rates (OCRs) illustrate the impacts of *ZFHX3* KD on mitochondrial function in the presence of (**A**) Etomoxir (CPT-1a inhibitor, 40 µM) and (**B**) UK5099 (mitochondrial pyruvate carrier inhibitor, 8 µM). Blue circles indicate control cells, and red triangles indicate *ZFHX3* KD cells. *ZFHX3* KD cells exhibited significantly higher acute response, maximal respiration, and spare capacity compared with control cells following Etomoxir injection (*n* = 6). Control and *ZFHX3* KD cells showed similar acute response, maximal respiration, and spare capacity in the presence of UK5099 (*n* = 6). ** *p* < 0.01.

We studied the effects of the *ZFHX3* gene on cellular energy substrate oxidation using specific inhibitors of substrate oxidation pathways. As shown in Figure 1, under low substrate demand, both control and *ZFHX3* KD cells had similar basal respiration. However, *ZFHX3* KD cells showed a significantly higher acute response, maximal respiration, and spare capacity after Etomoxir injection (a CPT-1a inhibitor that blocks FAs oxidation) as shown in Figure 1A. In contrast, after UK5099 injection, both control and *ZFHX3* KD cells displayed similar acute response and maximal respiration, along with a marked reduction in spare capacity as shown in Figure 1B. These findings suggest that *ZFHX3* KD increases FAs utilization but does not alter glucose utilization.

### 2.2. Effects of ZFHX3 Knockdown on Lactate Production, Glucose Uptake, and PDH Activity

**Figure 2:**
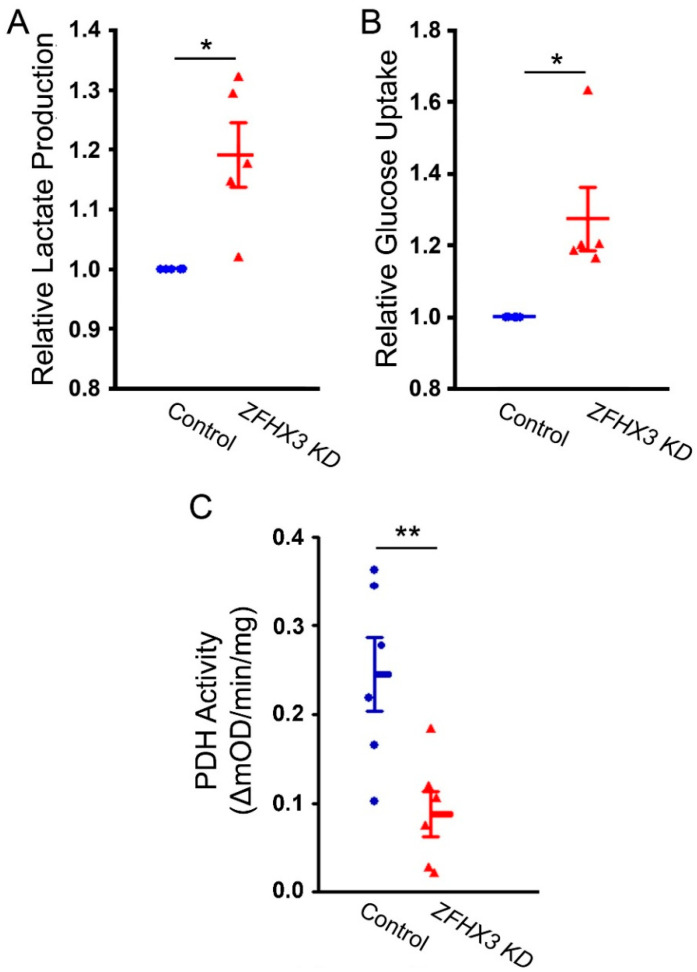
Effects of *ZFHX3* knockdown on lactate production, glucose uptake, and pyruvate dehydrogenase (PDH) activity. Average data of lactate concentrations (**A**) and glucose uptake (**B**) in control and *ZFHX3* KD HL-1 cells (*n* = 5). *ZFHX3* KD cells showed higher lactate concentrations and glucose uptake than control cells. (C) Average PDH enzyme activity in control and *ZFHX3* KD HL-1 cells, showing significantly lower PDH activity compared with control cells (*n* = 6). Blue circles represent control cells, and red triangles represent *ZFHX3* KD cells. * *p* < 0.05, ** *p* < 0.01.

To study the impact of increased FAs utilization on cellular metabolism, we examined lactate production, glucose uptake, and PDH activity. *ZFHX3* KD cells showed significantly higher lactate levels, along with increased glucose uptake and reduced PDH activity compared with control cells (Figure 2). These findings suggest that enhanced FAs utilization may suppress PDH activity, leading to an accumulation of lactate in *ZFHX3* KD cells.

### 2.3. Effects of ZFHX3 Gene on FAs and Glucose Metabolism Regulatory Proteins

**Figure 3:**
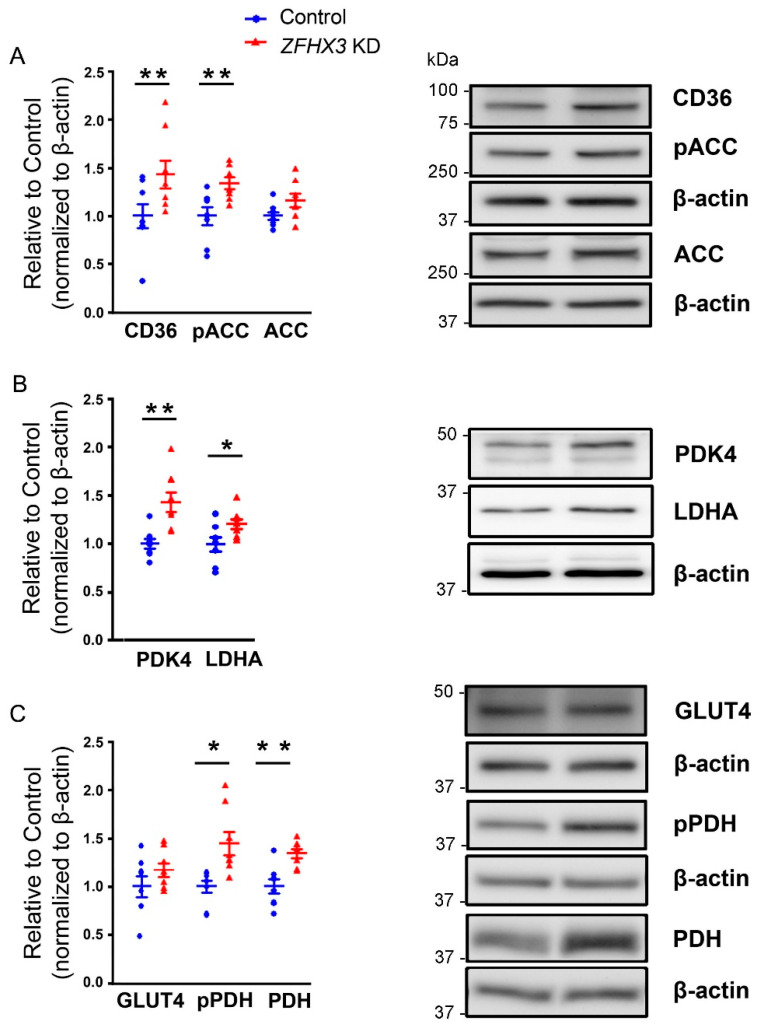
Effects of *ZFHX3* gene on fatty acids (FAs) and glucose metabolism regulatory proteins. (**A**–**C**) Representative Western blot and quantified data revealed a significant increase in CD36, phosphorylated ACC (pACC), pyruvate dehydrogenase kinase 4 (PDK4), lactate dehydrogenase A (LDHA), phosphorylated PDH (pPDH), and total PDH levels in *ZFHX3* KD HL-1 cells compared with control cells (*n* = 8). Blue circles represent control cells, and red triangles represent *ZFHX3* KD cells. * *p* < 0.05, ** *p* < 0.01.

We studied the regulatory proteins involved in FAs and glucose metabolism and found that *ZFHX3* KD cells exhibited higher expressions of CD36, phosphorylated ACC (pACC), phosphorylated PDH (pPDH), and total PDH proteins compared with control cells (Figure 3). However, both control and *ZFHX3* KD cells showed similar expressions of ACC and GLUT4.

### 2.4. Trimetazidine on Lactate Production

**Figure 4:**
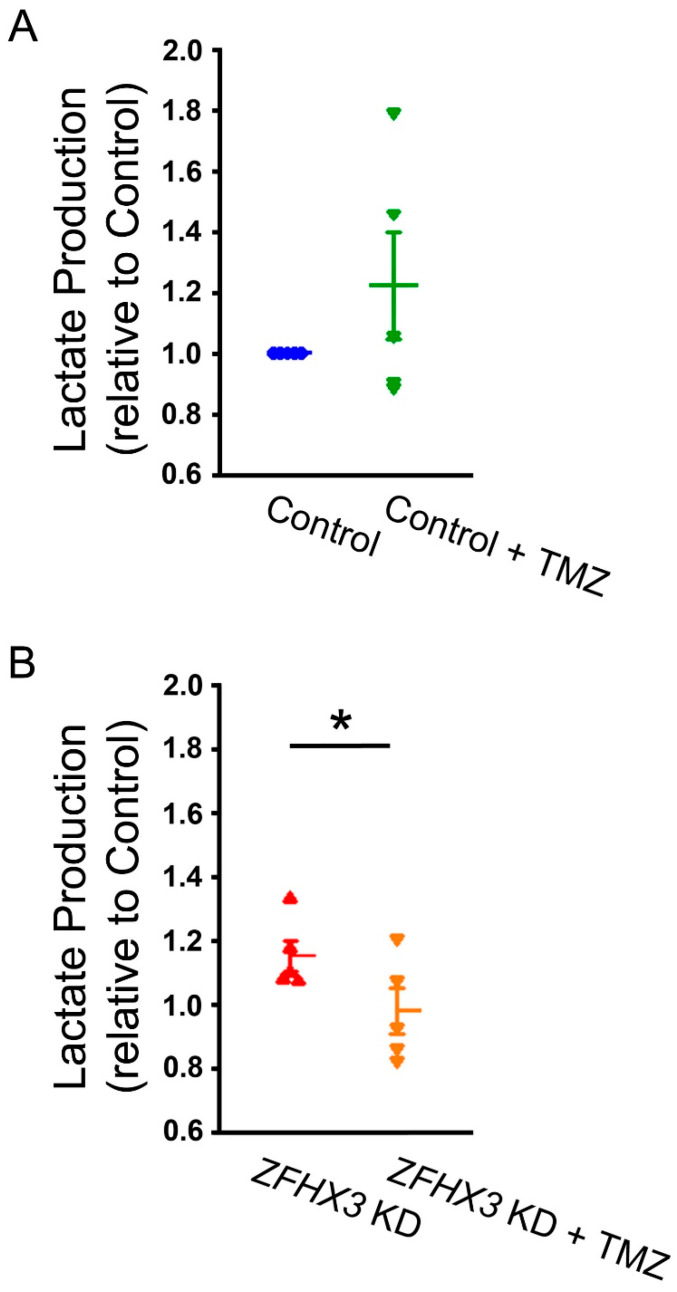
Effects of trimetazidine (TMZ) on lactate production. (**A**) TMZ (10 µM) did not change lactate content in control cells (*n* = 5). (**B**) TMZ (10 µM) significantly reduced lactate production in *ZFHX3* KD cells (*n* = 5). Blue circles represent control cells, green inverted triangles represent control + TMZ cells, red triangles represent *ZFHX3* KD cells, and orange inverted triangles represent *ZFHX3* KD + TMZ cells. * *p* < 0.05.

To explore the impact of increased FAs utilization on cardiac metabolism in *ZFHX3* KD cells, we studied the effect of trimetazidine (TMZ), a β-oxidation inhibitor, on lactate production in HL-1 cells. As shown in Figure 4, TMZ (10 µM)-treated *ZFHX3* KD cells exhibited lower lactate production compared with untreated *ZFHX3* KD cells. However, lactate levels were similar between control cells and TMZ-treated control cells.

### 2.5. Trimetazidine on Mitochondrial Respiration and Calcium Homeostasis

**Figure 5:**
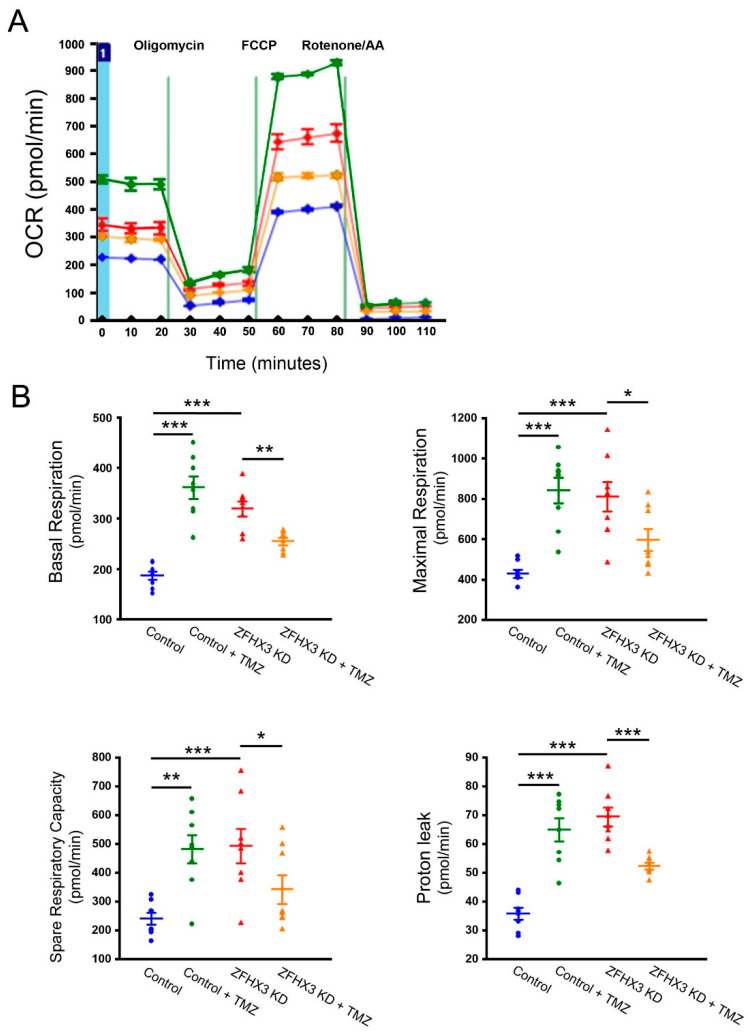
Effects of TMZ on oxygen consumption rates (OCRs) and bioenergetics parameters. (**A**) Representative traces and (**B**) quantified data illustrate the effects of TMZ (10 µM) on basal respiration, maximal respiration, spare respiratory capacity, and proton leak in control and *ZFHX3* KD cells, both with and without TMZ treatment (*n* = 8). Blue circles represent control cells, green circles represent control + TMZ cells, red triangles represent *ZFHX3* KD cells, and orange triangles represent *ZFHX3* KD + TMZ cells. * *p* < 0.05, ** *p* < 0.01, *** *p* < 0.005.

**Figure 6:**
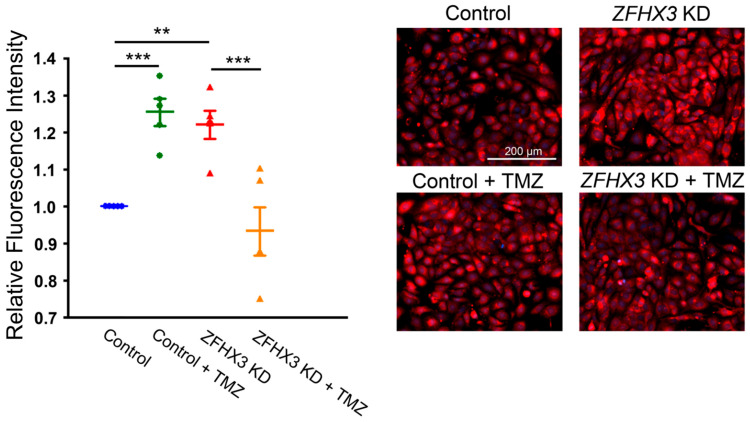
Effects of TMZ on mitochondrial Ca^2+^ content. Representative images and quantified data show the effects of TMZ (10 µM) on mitochondrial Ca^2+^ content in control (*n* = 5) and *ZFHX3* KD HL-1 cells (*n* = 5), with and without TMZ treatment. Blue circles represent control cells, green circles represent control + TMZ cells, red triangles represent *ZFHX3* KD cells, and orange triangles represent *ZFHX3* KD + TMZ cells. ** *p* < 0.01, *** *p* < 0.005 versus respective control groups.

**Figure 7:**
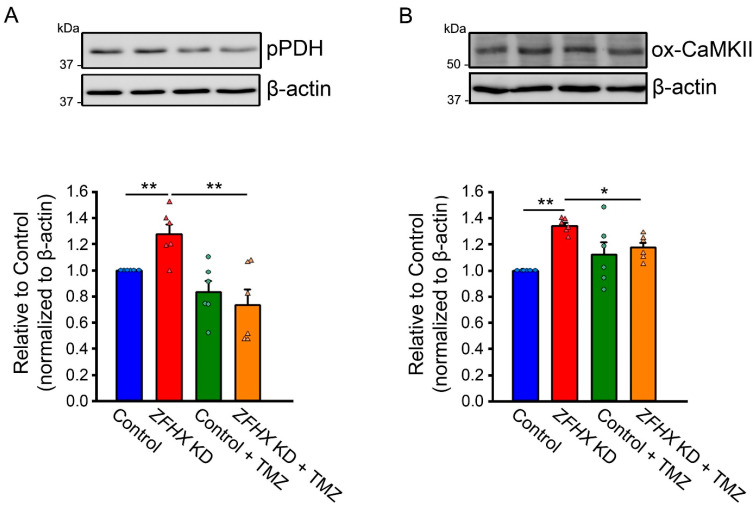
Effects of TMZ on pPDH and ox-CaMKII protein expressions. (**A**) phosphorylated PDH (pPDH) and (**B**) oxidized CaMKII (ox-CaMKII) protein levels were significantly decreased in *ZFHX3* KD HL-1 cells treated with TMZ (10 µM) compared with cells without TMZ treatment (*n* = 6). * *p* < 0.05, ** *p* < 0.01.

We next studied the effect of TMZ on *ZFHX3* KD-induced mitochondrial respiratory distress calcium homeostasis. TMZ (10 µM) reduced basal respiration by 20% and proton leak by 26% in *ZFHX3* KD cells (Figure 5). Conversely, TMZ increased basal respiration and proton leak in control cells. *ZFHX3* KD cells exhibited higher mitochondrial calcium content, measured using X-Rhod-1 fluorescence, compared with control cells. This increase was reduced by TMZ, whereas TMZ treatment led to increased mitochondrial calcium content in control cells (Figure 6). Furthermore, TMZ attenuated *ZFHX3* KD-induced increase in pPDH and oxidized CaMKII (ox-CaMKII) protein expression (Figure 7).

## 3. Discussion

Significant associations between AF and the *ZFHX3* gene have been recognized for over a decade, yet the molecular mechanisms by which this gene contributes to AF remain incompletely understood. Previous studies have highlighted substantial changes in cardiac energy metabolism, including mitochondrial dysfunction and altered protein levels responsible for both FAs and glucose oxidation pathways in left atrial appendage tissues from AF patients [4]. More recently, pathways such as oxidative phosphorylation and FAs metabolism were found to be downregulated in *ZFHX3*-deficient murine atria [15]. In the present study, we confirmed that *ZFHX3* KD cells had greater phosphorylated STAT3 expression (Supplementary Figure S1), which is consistent with previous findings [12,13] and is known to be a key regulator of FAs oxidation. Moreover, we demonstrated that *ZFHX3* KD in cardiomyocytes leads to dysregulation of both FAs and glucose metabolism. *ZFHX3* KD cells exhibited increased glucose uptake and lactate production, along with elevated LDHA protein expressions compared with control cells, indicating a shift toward glycolysis—a rapid, oxygen-independent but less efficient ATP-producing pathway [16,17,18]. Additionally, *ZFHX3* KD cells showed decreased PDH activity, with increased levels of both total and phosphorylated PDH and PDK4 proteins. PDK phosphorylates and inactivates PDH, a rate-limiting enzyme in glucose oxidation, thereby promoting glycolysis [2]. AF has been associated with increased lactate levels, downregulation of PDH, and enhanced PDK4 protein expressions in rabbits, consistent with increased glycolysis but reduced glucose oxidation in their hearts [19,20]. These findings suggest that *ZFHX3* KD results in uncoupling between glycolysis and pyruvate oxidation, confirming the presence of aerobic glycolysis (Warburg effect) in AF [21,22]. This metabolic shift leads to lactate accumulation and related metabolic abnormalities, which may contribute to dysregulated electrophysiological characteristics in atrial myocytes. Our study also found that *ZFHX3* KD cells exhibited higher protein expressions of CD36 and phosphorylated ACC (pACC), while total ACC remained unchanged. ACC is inactivated by phosphorylation and is a major downstream target of AMPK, which we previously found to be upregulated in *ZFHX3* KD cells [14]. AMPK is a primary energy sensor activated by metabolic stress in the heart [23]. In addition, STAT3 is implicated in the transcriptional regulation of CD36 [24], supporting the observed increase in CD36 expression in *ZFHX3* KD cells. Together, these findings support a shift toward FA uptake and utilization in ZFHX3-deficient atrial myocytes. Mechanistically, ZFHX3 has been reported to regulate STAT3 signaling, and downregulation of *ZFHX3* (ATBF1) can activate STAT3 via PIAS3 [13]. Activated STAT3 directly binds to the CD36 promoter, enhancing its transcription and thereby facilitating fatty acid uptake [24]. This pathway provides a plausible explanation for the observed CD36 upregulation in *ZFHX3* KD cells. In contrast, we did not find evidence that STAT3 directly regulates PDK4 or LDHA. Their upregulation may involve alternative transcriptional pathways or indirect effects. For example, AMPK activation enhances LDHA and PDK4 expression through metabolic stress signaling, while PPARα is known to regulate CD36 and PDK4 transcription via promoter binding. Although additional regulators or control compounds were not examined in this study, future work will investigate the roles of AMPK, PPARα, and related pathways in mediating these effects.

Elevated free FAs and their increased cellular uptake contribute significantly to atrial arrhythmogenesis. Previous studies have demonstrated that disrupted FAs metabolism—including FAs availability, uptake, transport, and oxidation—induces atrial metabolic inflexibility and promotes AF [2]. Upregulation of CD36, a key FAs transporter, and subsequent FAs overloading have been strongly correlated with AF [4] and its risk factors, such as obesity [25,26], diabetes [27], and aging [28]. Importantly, increased FAs uptake is not always matched by proportional FAs oxidation [29]. In our study, *ZFHX3* KD cells exhibited significantly higher acute response, maximal respiration, and spare capacity following Etomoxir injection compared with control cells, whereas both groups displayed similar responses to UK5099. Taken together, decreased PDH activity and heightened sensitivity to FAs oxidation inhibition suggest that *ZFHX3* KD cells rely more heavily on FA metabolism in the mitochondria. Excessive FA availability and β-oxidation increase mitochondrial oxygen demand and ROS production through enhanced electron transport chain activity and superoxide leakage [30,31]. Excessive ROS oxidizes CaMKII, a central regulator of Ca^2+^ handling, thereby impairing calcium homeostasis and promoting mitochondrial dysfunction, inflammation, and arrhythmogenesis [30,32,33]. Our previous study demonstrated that *ZFHX3* KD cells exhibited mitochondrial Ca^2+^ overload and elevated superoxide production [14]. We also reported that *ZFHX3* KD enhances IP3 receptor (IP3R) expression and signaling [14], promoting spontaneous SR Ca^2+^ release and excessive mitochondrial uptake. In addition, *ZFHX3* KD increased SERCA2a expression and activity, as well as RyR2 protein levels [12], collectively contributing to augment Ca^2+^ uptake and leak in atrial myocytes. These mechanisms, in addition to metabolic stress (lactate acidosis, ROS), establish multiple upstream pathways driving mitochondrial Ca^2+^ overload in *ZFHX3*-deficient cells. Other calcium-handling proteins may also play roles in ZFHX3 deficiency-mediated calcium dysregulation and warrant further investigation.

TMZ is a clinically used anti-angina agent that inhibits FAs oxidation, thereby reducing oxidative stress and oxygen demand [34,35]. Additionally, TMZ has been suggested as a potential therapeutic agent in heart failure through metabolic regulations [36,37]. In this study, TMZ significantly reduced mitochondrial respiration and proton leak in *ZFHX3* KD cells. The greater effect on proton leak compared with basal respiration suggests that inhibiting FAs utilization helps preserve mitochondrial function under stress. TMZ also reduced lactate production in *ZFHX3* KD cells, supporting the concept that increased FAs utilization drives lactate accumulation and exacerbates mitochondrial Ca^2+^ overload. Interestingly, TMZ exhibited differential effects in control versus *ZFHX3* KD cells. In control cells, TMZ increased mitochondrial respiration and calcium content, likely due to enhanced ATP synthesis under normal conditions, whereas in ZFHX3 KD cells, TMZ exerted protective effects by reducing mitochondrial proton leak, lactate accumulation, and mitochondrial Ca^2+^ overload. This suggests that TMZ’s impact is context-dependent, mitigating metabolic distress in *ZFHX3* KD cells while potentially enhancing mitochondrial function in healthy cells. Mechanistically, TMZ reduced PDH phosphorylation in *ZFHX3* KD cells, improving glucose utilization, and lowered ox-CaMKII levels, suggesting that FAs inhibition mitigates metabolic stress-induced calcium dysregulation. Notably, at higher concentrations (10–100 μM), TMZ has been shown to enhance mitochondrial calcium uptake, supporting ATP synthesis [38]. In summary, *ZFHX3* KD induces cardiac metabolic stress characterized by decreased PDH activity and increased β-oxidation, leading to mitochondrial dysregulation (calcium overload, proton leak), lactate accumulation, and enhanced ox-CaMKII, all of which may contribute to electrical remodeling and arrhythmogenesis. However, there are several limitations of our study that must be acknowledged. Our study was conducted in HL-1 atrial myocytes, a well-established in vitro model. While this system enabled mechanistic dissection of *ZFHX3* KD effects under controlled conditions, the results may not fully reflect the complexity of primary cardiomyocytes or in vivo AF models. Future studies using animal models and patients-derived cells are needed to validate these findings. In addition, we examined the global effects of *ZFHX3* knockdown but did not investigate specific *ZFHX3* variants, which may have distinct impacts on cardiac metabolism and AF pathology. Finally, our findings are specific to atrial myocytes and may not directly apply to ventricular cardiomyocytes, which can display different metabolic and calcium-handling properties. *ZFHX3* KD induces cardiac metabolic stress characterized by decreased PDH activity and increased FA oxidation, leading to mitochondrial dysfunction (proton leak, calcium overload), lactate accumulation, and enhanced ox-CaMKII. These alterations contribute to electrical remodeling and arrhythmogenesis. TMZ mitigates these effects, highlighting its therapeutic potential as a metabolic modulator in AF associated with ZFHX3 deficiency.

## 4. Materials and Methods

### 4.1. HL-1 Cell Culture and Lentiviral shRNA-Mediated ZFHX3 Knockdown

HL-1 cardiomyocytes (kindly provided by Dr. Claycomb, Louisiana State University Medical Center, New Orleans, LA, USA) were cultured in Claycomb medium (JRH Biosciences, Lenexa, KS, USA, Cat. No. 51800C) supplemented with 10% fetal bovine serum (FBS, JRH Biosciences, Cat. No. 97068-085), in flasks pre-coated with fibronectin (Sigma (St. Louis, MO, USA), Cat. No. F1141) and gelatine (Sigma, Cat. No. G9391) [14]. Cells were incubated at 37 °C in a 5% a CO_2_ incubator (Thermor (Saint-Jean-de-la-Ruelle, France), Model 3111). *ZFHX3* knockdown was achieved using lentivirus containing shRNA targeting *ZFHX3* (clone TRCN0000321285, National RNAi core Facility, Academia Sinica, Taipei, Taiwan). Cells were selected with puromycin (Sigma, Cat. No. P8833) at 2 µg/mL for 1 week and subsequently maintained at 1 µg/mL.

### 4.2. Intracellular Lactate Concentrations

To measure intracellular L-lactate concentrations, a colorimetric Lactate assay kit (Cat. No. MAK064, Sigma) was used according to the manufacturer’s instructions. HL-1 cells were plated into a 6-well plate and allowed to attach and normalize functionally overnight. The cells were maintained in buffered culture medium containing 10% FBS and were allowed to reach approximately 90% confluence. Cells were then treated with or without TMZ (10 µM, Sigma) for 3 h. Cells were trypsinized and the resulting cell pellet was processed according to the kit protocol. Briefly, the cell pellet was homogenized in 4 volumes of lactate assay buffer, then centrifuged at 13,000× *g* for 10 min to remove insoluble material. The supernatant was deproteinized using a 10 kDa MWCO spin filter to remove endogenous lactate dehydrogenase. The soluble fraction was then assayed directly.

### 4.3. PDH Activity Assay

The catalytic activity of PDH was measured using the PDH Enzyme Activity Microplate Assay Kit (ab109902, Abcam (Cambridge, UK)) according to the manufacturer’s instructions. Cells were plated in a 6-well plate and allowed to attach and normalize functionally overnight. After washing with PBS and trypsinizing, the cell pellet was processed as per the kit instructions. Briefly, the fresh cell pellet was resuspended in 9 volumes of PBS, and proteins were extracted by adding the detergent solution. Freshly prepared samples with a concentration of 100 µg/200 µL were loaded into microplate wells coated with an anti-PDH monoclonal antibody and incubated for 3 h at room temperature. PDH activity was determined by measuring the rate of NAD^+^ reduction to NADH, coupled with the reduction in a reporter dye, and monitoring the increase in absorbance of the reaction product at 450 nm. All tests were conducted in triplicate, and results were expressed as changes in absorbance per minute per milligram of protein.

### 4.4. Seahorse Substrate Oxidation Stress Test and Mitochondrial Oxygen Consumption Rate (OCR)

For mitochondrial OCR measurements, HL-1 cells were first cultured under standard conditions in general culture plates. On the day of the Seahorse assay, cells were detached and seeded at a density of 2 × 10^5^ cells/well in unbuffered Dulbecco’s Modified Eagle Medium (DMEM, pH 7.4) into an XFe24 Seahorse assay plate (Seahorse Bioscience, Billerica, MA, USA). The plates were incubated at 37 °C in a non-CO_2_ incubator for 45–60 min to allow temperature and pH equilibration. The Seahorse sensor cartridge was hydrated overnight at 37 °C in a non-CO_2_ incubator with sterile deionized water.

To evaluate mitochondrial substrate oxidation, we utilized the XF24 LCFA Oxidation Stress Test (Cat# 103672-100, Agilent Technologies, Santa Clara, CA, USA) and the XF24 Glucose/Pyruvate Oxidation Stress Test (Cat# 103673-100, Agilent Technologies). The assay media compositions were as follows.

LCFA Oxidation Stress Test (Figure 1A): 0.5 mM D-glucose, 1 mM L-glutamine, 0.5 mM L-carnitine, and 1% FBS. Glucose/Pyruvate Oxidation Stress Test (Figure 1B): 1 mM sodium pyruvate, 2 mM L-glutamine, and 25 mM D-glucose. Mito Stress Test (Figure 5): identical to the Glucose/Pyruvate Oxidation Test medium; in addition, cells were treated with or without trimetazidine (TMZ, 10 µM; Sigma) for 3 h prior to the assay.

OCR was measured sequentially following injections of the following compounds: Etomoxir (40 µM) to inhibit fatty acid oxidation via CPT-1a, or UK5099 (8 µM) to inhibit mitochondrial pyruvate carrier, followed by oligomycin (1.5 µM) to inhibit ATP synthase, FCCP (1 µM) to uncouple oxidative phosphorylation and induce maximal respiration, and finally, rotenone (0.5 µM) plus antimycin A (0.5 µM) to inhibit complexes I and III, thereby measuring non-mitochondrial respiration.

Absolute OCR values (in pmol/min) were recorded at each injection step. The following parameters were calculated using standard Seahorse equations. Basal respiration: Initial OCR prior to any drug injection (absolute value). Maximal respiration: FCCP-stimulated OCR minus non-mitochondrial OCR (absolute value). Spare respiratory capacity: Maximal OCR minus basal OCR (calculated delta). Acute response: OCR immediately after substrate inhibitor injection minus baseline OCR (calculated delta). Proton leak: OCR after oligomycin minus non-mitochondrial OCR (calculated delta).

### 4.5. Glucose Uptake

Glucose uptake of cardiomyocytes was measured using the Glucose Uptake-Glo^TM^ (Promega, Madison, WI, USA) bioluminescence assay kit following the manufacturer’s instructions. After washing the cells twice with 1× PBS, 500 µL of 1 mM 2-DG (2-deoxyglucose) diluted in PBS was added for a 10 min uptake period. Following uptake, stopping and neutralization buffers were applied and 2-deoxyglucose-6-phosphate detection reagent were added. Luminescence intensity was recorded at 1 h using a Tecan Infinite M200 plate reader.

### 4.6. Mitochondrial Ca^2+^ Measurements

Mitochondrial Ca^2+^ levels were assessed in intact live cells using fluorescence microscopy (EVOS FL, Invitrogen (Waltham, MA, USA)). Cells were treated with or without TMZ (10 µM, Sigma) for 3 h. After treatments, cells were stained with 2.5 μM X-Rhod-1/AM for 10 min at 37 °C, followed by a 10 min incubation with 1 mM CoCl_2_ in Tyrode’s solution to quench cytosolic X-Rhod-1 fluorescence.

### 4.7. Western Blotting

Control and *ZFHX3* KD HL-1 cells with or without TMZ (10 µM, Sigma) for 3 h were lysed in M-PER Mammalian Protein Extraction Reagent (Thermo Fisher Scientific, Cat. No. 78501) with protease inhibitor cocktails (Sigma, St. Louis, MO, USA). Equal amounts of proteins from each group were separated in gradient sodium dodecylsulphate polyacrylamide gel electrophoresis under reducing conditions and electrophoretically transferred onto an equilibrated polyvinylidene difluoride membrane (Amersham Biosciences, Amersham, Buckinghamshire, UK). Blots were probed with following primary antibodies: ACC (1:1000, Abcam, Cat. No. ab6564), GLUT4 (Abcam, ab6564), CD36 (Santa Cruz Biotechnology, Dallas, TX, USA), PDH (Cell Signaling, Danvers, MA, USA), pPDH (Serine293, Sigma), pACC (#07303, Sigma), PDK4, LDHA, SLC16A1 (Antibodies.com), and oxidized CaMKII (Genetex, Irvine, CA, USA). An enhanced chemiluminescence detection system (Santa Cruz Biotechnology, Santa Cruz, CA, USA) was used to detect bound antibodies, which were then analyzed using AlphaEase FC software version 4.0. (Alpha Innotech, San Leandro, CA, USA). Targeted bands were normalized to β-actin (Antibodies) to confirm equal protein loading.

### 4.8. Statistical Analysis

All quantitative data are expressed as the mean ± standard error of the mean (SEM). Differences between groups were assessed using an unpaired test while comparisons among groups were made using two-way analysis of variance with repeated or non-repeated measures, followed by Turkey’s post hoc test where appropriate. Statistical significance was set at *p* < 0.05.

## Data Availability

Data is contained within the article or Supplementary Material: The original contributions presented in this study are included in the article/Supplementary Material. Further inquiries can be directed to the corresponding author.

## Supplementary Materials

The following supporting information can be downloaded at https://www.mdpi.com/article/10.3390/ijms26178576/s1.
