# Supplementary material for: ZFHX3 Knockdown Enhances Metabolic Distress in Atrial Myocytes Through Mitochondrial and Calcium Dysregulation: Mitigation by Trimetazidine
Source: Int J Mol Sci. 2025 Sep 3;26(17):8576. doi: 10.3390/ijms26178576 (PMC12429405; doi:10.3390/ijms26178576)
Supplement: Supplementary file 1 [file ijms-26-08576-s001.zip › ijms-3810371-supplementary.pdf]

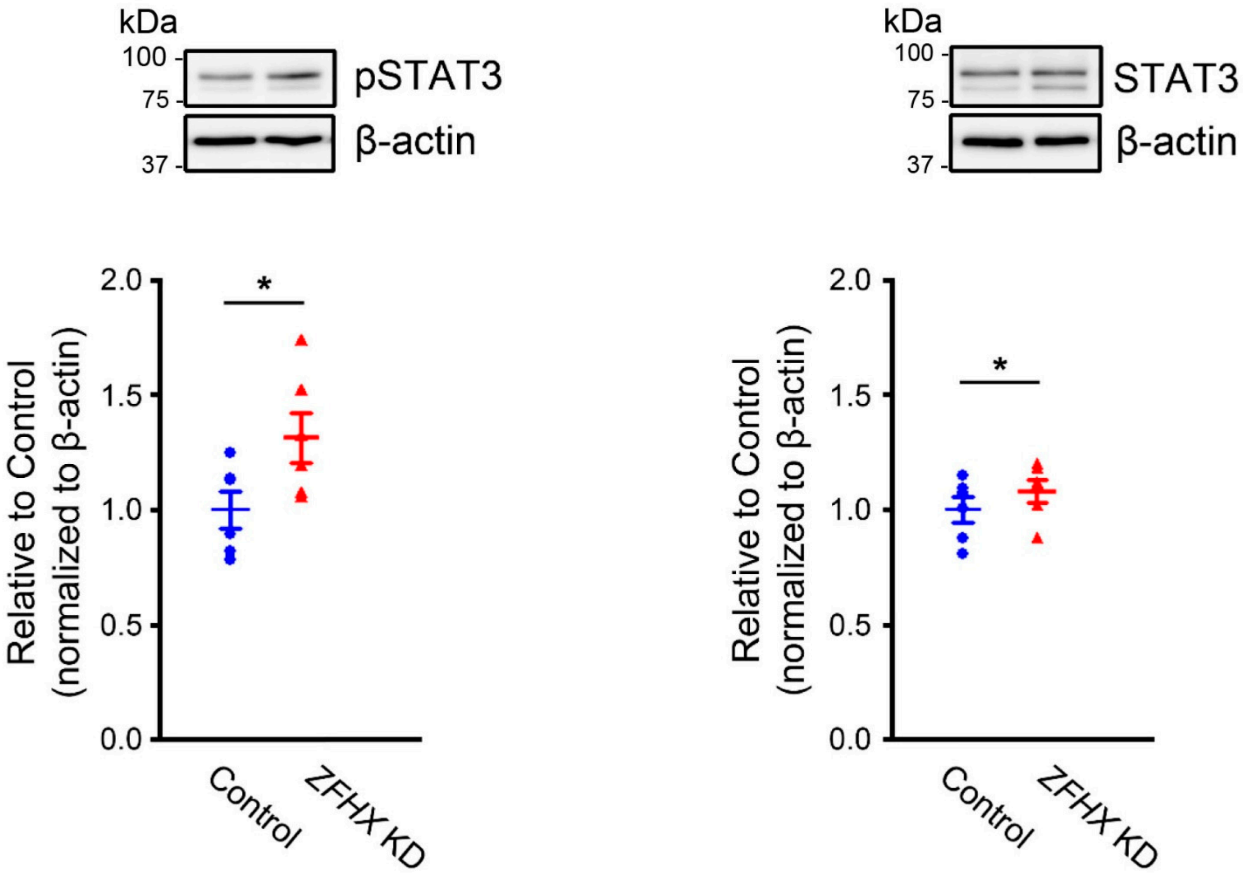

Supplementary Figure S1. The expression levels of pSTAT3/STAT3 proteins in control and ZFHX3 KD cells. ZFHX3 KD cells had a higher expression of phosphorylated and total STAT3 proteins than control cells ($n = 6$). *$p < 0.05$. Control and ZFHX3 KD HL-1 cells were lysed in protein extraction reagent (Thermo Fisher Scientific) with protease inhibitor cocktails (Sigma, St. Louis, MO, USA). Equal amounts of proteins from each group were separated in gradient sodium dodecylsulphate polyacrylamide gel electrophoresis under reducing conditions and electrophoretically transferred onto an equilibrated polyvinylidene difluoride membrane (Amersham Biosciences, Buckinghamshire, UK). Blots were probed with primary antibodies: pSTAT3 (Cell Signaling) and STAT3 (Millipore). An enhanced chemiluminescence detection system (Santa Cruz Biotechnology, Santa Cruz, CA, USA) was used to detect bound antibodies, which were then analysed using AlphaEase FC software (Alpha Innotech, San Leandro, CA, USA). Targeted bands were normalized to β-actin to confirm equal protein loading.
